# Innovative Perspectives on Phytochemicals in Human Nutrition

**DOI:** 10.3390/nu17040711

**Published:** 2025-02-17

**Authors:** Valentina Melini, Maurizio Ruzzi

**Affiliations:** 1 CREA Research Centre for Food and Nutrition, Via Ardeatina 546, I-00178 Rome, Italy; 2 Department for Innovation in Biological, Agro-Food and Forest Systems (DIBAF), University of Tuscia, I-01100 Viterbo, Italy

## Editorial on the Research Topic “Innovative Perspectives on Phytochemicals in Human Nutrition”

Phytochemicals are chemical compounds naturally occurring in plants [1]. Some are required for plant growth, while others mediate interactions between plants and the environment [2]. For example, they can protect plants from disease and damage and contribute to plant tissues’ color, aroma, and flavor. Because of their varied structures, phytochemicals have not yet been strictly categorized [1]. Phenolic compounds, carotenoids, alkaloids, and terpenes are some of the most significant phytochemicals. Phenolic compounds present one or more hydroxyl groups on the aromatic ring(s) and play a pivotal role in plants’ development and defense mechanisms, thus, contributing to their resilience and adaptability to environmental challenges. Carotenoids consist of eight isoprenoid units, and their conjugated C=C chemical bonds are responsible for the red/orange/yellow color of plant tissues; they, thus, play a pivotal role in attracting insects for pollination. Alkaloids include phytoalexins that are biosynthetically derived from the amino acid (S)-tryptophan and contain sulfur; they protect plants from pathogens. Terpenes interact with free radicals to exert an antioxidant function. Phytochemicals have antimicrobial, antiviral, antidiarrheal, anthelmintic, antiallergic, and antimicrobial activity and significantly impact human health [3]. Vegetables, fruits, whole grains, nuts and seeds, and spices are rich in phytochemicals and are the main dietary source thereof; their daily consumption has been reported to reduce risk for non-communicable chronic diseases such as cardiovascular diseases, type 2 diabetes, and some types of cancer.

The content of phytochemicals in foods and their effect on human health has been extensively studied in the last two decades. From 2000 to 2024, the number of papers dealing with phytochemical content and bioactivity increased from 48 to 2314 in the Scopus database. Many papers report on the phytochemical content in foods, thus, providing important data enabling the estimation of phytochemical dietary intake. Recently, research has been spurred by interest in understanding the effects of food processing, including storage conditions and cooking, on phytochemicals.

However, many aspects about the role of these components in human nutrition are still underexplored and the definition of recommended dietary intake of phytochemicals remains a challenge. Insights into the relationship between phytochemical structures and their function are also important, as well as their bioaccessibility and bioactivity.

The aim of this Special Issue was to gather research articles and review papers providing innovative perspectives on the contribution of phytochemicals to health and well-being promotion.

A total of six papers were published within the research topic “Innovative Perspectives on Phytochemicals in Human Nutrition”: two reviews and four research articles.

The review by Küçükgöz and Trząskowska (contribution 1) provides an overview of raw materials and strains that might be used for producing non-dairy probiotic products. So far, probiotics are mainly supplied by dairy products. However, the latter pose nutritional and ethical issues. The occurrence of cholesterol and lactose makes them inadequate for people suffering from cardiovascular diseases or people who are lactose-intolerant, respectively. Moreover, people who adhere to vegan diets do not consume dairy products. Hence, plant foods containing probiotics might represent a choice that meets the requirements for healthy, ethical, and sustainable diets. The need to intensify research on developing non-dairy probiotic foods and the need to assess their nutritional quality by in vitro models was stressed.

The paper by Halal et al. revised the biological activity of thyme (*Thymus vulgaris* L.) (contribution 2), one of the most popular wild edible plants native to the Mediterranean region. The composition of thyme in terms of phytonutrients, minerals, vitamins, flavonoids, and antioxidants is reviewed. Moreover, the therapeutic use of thyme and its essential oils, especially rich in thymol and carvacrol, is presented.

Two research papers deal with culinary herbs (contribution 3, contribution 4). The paper by Vita et al. (contribution 3) investigates the associations between the frequency of culinary herb consumption and gut microbiota. The study, thus, contributes to filling in the gap of current research on the interactions between diet and the human gut microbiome, which commonly excludes an investigation on culinary herbs. It emerged that the frequency of spice use is related to the diversity of the microbiome, and the abundance of specific taxa. Indeed, after adjusting for dietary factors, the total number of spices used over three times per week was positively associated with *Firmicutes* abundance and negatively associated with *Proteobacteria* abundance. In contrast, the frequency of culinary herb use was not associated with *Bacteroidota* or *Actinobacteria* abundance. The paper by Martinelli et al. (contribution 4) investigates the nutraceutical properties of Sumac (*Rhus coriaria* L.) against gastritis. Sumac is spread in the Mediterranean region and in Middle East, and can be used as a spice and medicinal herb. The research group investigated the effect of several polar extracts in gastric epithelial cells challenged by TNF-α or *Helicobacter pylori* (*H. pylori*) infection. The ethanolic extracts exhibited the greatest phenolic contents, which correlate with lower half maximal inhibitory concentrations (IC50s) on the release of interleukin-8 and interleukin-6 induced by TNF-α. Moreover, the extracts also inhibited the release of IL-8 during *H. pylori* infection (contribution 4).

Two research papers used animal models to investigate the biological activities of phytochemicals (contribution 5, contribution 6). The paper by Huang et al. investigated the effect of *Citrus junos* Tanaka extracts on PM10-induced lung damage in BALB/c mice (contribution 5). It emerged that *C. junos* Tanaka significantly attenuated PM10-induced pulmonary damage and inflammatory cell infiltration in a mouse model, possibly because of their anti-inflammatory and antioxidant properties. Since the determination of antioxidant content is propaedeutic to the evaluation of the biological activity, the total soluble phenol and flavonoid contents were determined spectrophotometrically. In addition, naringenin and hesperidin were quantified. In the paper by Rocha et al. (contribution 6), an experimental model was used to study the effects of fish oil supplementation in rats with hypercholesterolemia induced by a hypercholesterolemic diet (HD). It was found that the mouse group fed with fish oil showed significantly lower plasma concentrations of triglycerides and hepatic myeloperoxidase and higher erythrocyte superoxide dismutase activity (*p* < 0.05). Moreover, it was observed that supplementation with fish oil also promoted hepatocyte reorganization and an expressive reduction in lipid vacuoles and hepatic triglyceride content.

Collectively, the articles included regarding this research topic provide an overview of different aspects related to food phytochemicals ranging from innovative products such as non-dairy probiotic products to the health effects of several spices.
